# Propagation of Subseasonal Equatorially-Forced Coastal Trapped Waves down to the Benguela Upwelling System

**DOI:** 10.1038/s41598-019-41847-1

**Published:** 2019-03-28

**Authors:** Serena Illig, Marie-Lou Bachèlery

**Affiliations:** 1 Laboratoire d’Etudes en Géophysique et Océanographie Spatiale (LEGOS), CNRS/IRD/UPS/CNES, Toulouse, France; 2 Department of Oceanography, MARE Institute, LMI ICEMASA, University of Cape Town, Rondebosch, South Africa; 3 Nansen-Tutu Centre, Marine Research Institute, Department of Oceanography, University of Cape Town, Rondebosch, South Africa

## Abstract

The oceanic connection between the coastal variability along the southwestern African coasts and the linear equatorial dynamics at subseasonal time-scales (<120 days) is examined using a variety of model outputs, ranging from linear to general circulation models. We focus on the equatorially-forced fast and weakly dissipative first-mode coastal trapped waves which are shown to propagate down to the southern tip of Africa. In the eastern equatorial Atlantic, the first-mode equatorial forcing is tangled with the higher-order Kelvin wave modes and is overshadowed by the dominant second baroclinic mode. The latter is slower and peaks 10 days after the concealed first-mode contribution. Within this time frame, the remotely-forced first-mode coastal trapped waves impinge on the variability of the Benguela upwelling ecosystem, almost in phase with the subseasonal sea level fluctuations in the Gulf of Guinea. Over 1993–2008, the equatorial forcing undergoes a substantial interannual modulation. Periods of energetic first-mode equatorial Kelvin waves coincide with a strong subseasonal coastal wind activity that breaks the stronger equatorial connection. This suggests the existence of a large-scale atmospheric connection between the equatorial wave forcing and the along-shore winds in the Benguela, modulating the maximum latitude at which the equatorial dynamics impacts the local marine resources.

## Introduction

**Figure 1:**
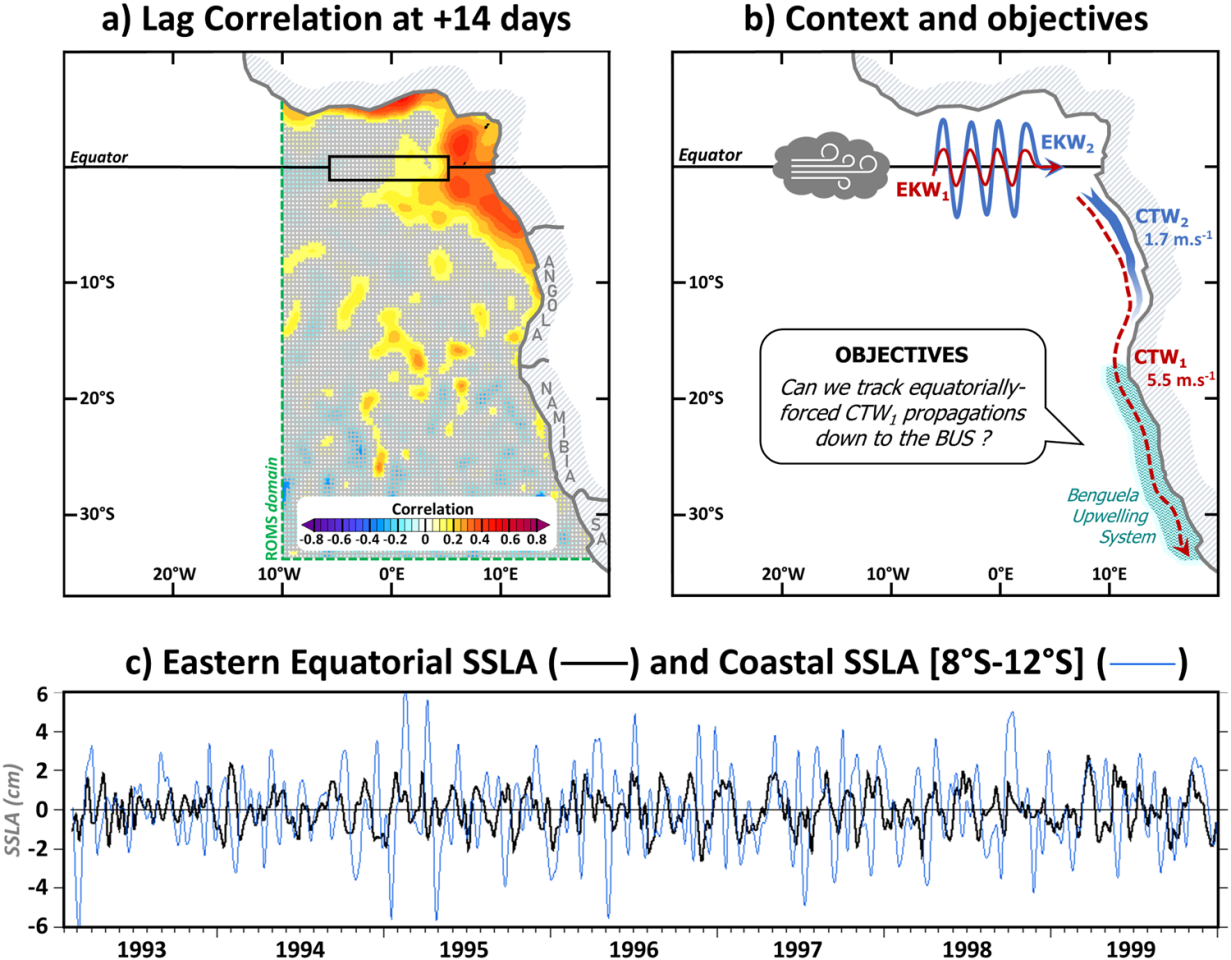
Context of the study. (**a**) Lag-correlation between SSLA in the eastern equatorial Atlantic (averaged within [5°W–5°E; 1°S–1°N]) and Subseasonal Sea Level Aanomalues (SSLA) in the Southeastern Atlantic taken two weeks later. The analysis is performed using AVISO altimetric data^19^ over the 1993–2008 period. Dashed green lines delineate our regional ocean model domain. (**b**) Schematic illustrating the findings of *Illig et al*.^6^ (*viz*. the dominance of the subseasonal second EKW mode in the eastern equatorial basin and the dissipation of the equatorially-forced subseasonal second CTW mode at ~12°S) and the objectives of the paper. Theoretical phase speed of CTW_1_ (averaged within [5°S–27°S]) and CTW_2_ (averaged within [5°S–12°S]) are estimated from Fig. 4 in *Illig et al*.^5^. (**c**) 1993–1999 timeseries of eastern equatorial (averaged within [5°W–5°E; 1°S–1°N]) and coastal (averaged within the 1°-width coastal band and between 8°S–12°S) altimetric SSLA (cm). (**a**,**c**) have been realized using the Ferret program (http://ferret.pmel.noaa.gov/Ferret/). (**b**) has been realized using Microsoft PowerPoint (https://products.office.com/fr/powerpoint).

The ocean dynamics along the coastal fringe of the southeastern Atlantic Ocean is affected by the remote linear equatorial variability at frequencies ranging from sub-monthly to interannual time-scales^1–6^. Upon reaching the coast of Gabon off West Africa, part of the eastward propagating long equatorial wave energy is transmitted southward along the southwestern coast of Africa as Coastal Trapped Waves (CTW)^7–9^, where along-shore wind fluctuations also excite poleward-propagating CTW^10^. Remotely- and locally-forced CTWs imprint the coastal sea level variability and can be detected from altimetry^1,11,12^. They also trigger substantial thermocline, halocline, and nutricline displacements, impinging on the West African coastal variability from the Congo polewards to the very productive Benguela Upwelling System (17°S–33°S; BUS; cf. Fig. 1b)^3,13,14^.

However, studies based on remote sensing data^1,15^ and high-resolution numerical models^2,6^, showed that at subseasonal time-scales (~2–120 days, Fig. 1c), the equatorial connection does not reach the northern BUS. As an illustration, Fig. 1a highlights that the coherence between the subseasonal equatorial fluctuations and the Subseasonal Sea Level Anomalies (SSLA) 2 weeks later fades out at ~12°S. In contrast, in the southeastern Pacific, the remotely-forced subseasonal signal can be detected along the coasts of Peru and Chile as far as ~30°S^6,16–18^. *Illig et al*.^6^ suggested that the difference between the two systems can be attributed to the difference in the vertical structure along the equatorial waveguides. For the 2000–2008 period, they reported that in the Humboldt system, the coastal subseasonal remotely-forced variability is controlled by the first CTW mode (CTW_1_), triggered by the dominant first baroclinic mode in the eastern equatorial Pacific. This mode is fast (~3.6 m.s^−1^ in southeastern Pacific^5^ and ~5.5 m.s^−1^ in the southeastern Atlantic^5^) and weakly dissipative, enabling a consistency with the equatorial variability at high latitudes. Conversely, in the southeastern Atlantic, the linear equatorial variability is dominated by the second Equatorial Kelvin Wave (EKW) mode which is transmitted along the coast of southwestern Africa as slower second-mode CTWs (~1.7 m.s^−1^ within [5°S–12°S]^5^). This is schematized in Fig. 1b. These coastal waves undergo stronger dissipation and scattering compared to that of first-mode CTWs. Their amplitudes drastically decrease south of ~12–15°S, where energetic locally wind-forced first-mode CTWs overshadow the remote signal.

In this study, we focus on the equatorial connection associated with the propagation of the first EKW mode (EKW_1_), because it is fast and weakly-dissipative when transmitted along the coast of southwestern Africa and can propagate farther south than the higher-order modes (as illustrated on Fig. 1b). We document its interannual modulation, in order to identify periods in which the EKW_1_ subseasonal activity is enhanced and may favor a strong connection with the equatorial variability that can be depicted in the BUS. Using outputs from a range of models of different complexity (from equatorial linear model to ocean general circulation models) along with altimetric observations, we examine the remote equatorial forcing characteristics and the impact of the coastal wind forcing. This brings us to reconsider the timing of the equatorial connection in the BUS and highlights the necessity to decipher the contribution of individual EKW and CTW modes.

## Data and Methods

We focus on the 1993–2008 period, over which the oceanic connection is examined using the AVISO 1/4° gridded maps of altimetric Sea Level Anomalies (SLA)^19^. The surface wind forcing is described using the DRAKKAR Forcing Set (DFS) v5^20^.

Subseasonal fluctuations are estimated as the departure from the monthly 1-2-1 weighted average time-series^15^. The difference between the original time-series and the subseasonal component isolates the summed-up contribution of the seasonal and interannual signals, which in this paper constitutes the low-frequency component. For the 1993–2008 subseasonal time-series used in this study, the threshold of the 99% significance correlation^21^ is 0.2.

5-day averages of SODA_2.1.6 reanalysis^22^ outputs are used to quantify the subseasonal equatorial forcing and its low-frequency modulation. Comparisons against *in-situ* PIRATA^23^ observations and remotely-sensed data from AVISO^19^ and Globcurrent^24^ (see Section S1 in supplementary material) indicate that SODA is skillful in simulating most aspects of the mean state and the subseasonal variability along the eastern equatorial wave-guide. Following the methodology developed in *Illig et al*.^25^ and *Dewitte et al*.^26^, the equatorial baroclinic structures are estimated from low-frequency and zonally slow-varying stratification. To extract the contributions of the gravest EKW modes, pressure and zonal current anomalies are projected onto the vertical structures and subsequently onto the EKW meridional structures. EKW are expressed in terms of their contribution to the equatorial Subseasonal SLA (SSLA).

SODA EKW contributions are compared to the solution of the equatorial Atlantic Ocean Linear Model (OLM) developed by *Illig et al*.^25^. This model simulates the linear propagation of long equatorial Kelvin and Rossby waves for the six gravest baroclinic modes, using wind-stress and wave parameters (phase speed, dissipation, and wind-projection coefficient) derived from SODA.

CTW contributions are estimated using the outputs of the ROMS^27^ v3.1 southeastern Atlantic configuration (34°S–7°N, 10°W-African coast; depicted in Fig. 1a) developed in *Bachèlery et al*.^28^. This configuration (ROMS^CR^) closely resembles the one of *Bachèlery et al*.^2^ and *Illig et al*.^5,6^, with a horizontal resolution of 1/12°, 37 sigma vertical levels and open lateral boundary conditions provided by SODA. In this study, the surface forcing consists of daily maps from DFS. Section S2 of the supplementary material provides a brief comparison between ROMS^CR^ outputs and observations. It shows that the linear equatorial dynamics is adequately constrained by SODA boundary forcing in the regional model and it is successfully transmitted along the coast of west Africa at subseasonal time-scales. To isolate the signature of the oceanic equatorial connection from the effects of the coastal atmospheric forcing, a sensitivity experiment (ROMS^EQ^) was performed (after *Illig et al*.^6^). Outside of the Gulf of Guinea, ROMS^EQ^ is forced by the low-frequency component of the surface forcing. Assuming some linearity, ROMS^EQ^ subseasonal coastal variability is only impacted by the equatorial variability, while in ROMS^CR^, remote and local forcings are concomitantly at work. 5-day averaged outputs of ROMS^CR^ and ROMS^EQ^ simulations are analyzed over the 1993–2008 period.

CTW modal structures of the 3 gravest CTW modes are derived using ROMS^CR^ mean stratification and topography^29^, over which subseasonal ROMS^CR^ and ROMS^EQ^ pressure anomalies are projected^5^. CTWs are expressed in terms of their contribution to coastal SSLA.

## Results and Discussion

### First-mode CTW propagates down to the BUS

**Figure 2:**
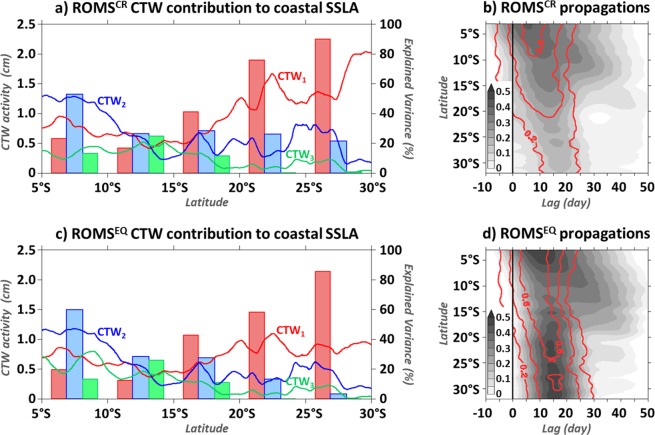
Mean (1993–2008) subseasonal CTW (averaged within the 0.5°-width coastal band) characteristics. (**a**) ROMS^CR^ CTW mode contribution to coastal SSLA. Red, blue and green plain lines show the RMS (cm) of CTW modes 1, 2, and 3, respectively, as a function of the latitude along the southwestern African coast. Red, blue and green bar-charts quantify the explained variance of CTW modes 1, 2 and 3 relative to the coastal SSLA. Time series are averaged in 5°-width latitudinal boxes ([5°S–10°S], [10°S–15°S], [15°S–20°S], [20°S–25°S], and [25°S–30°S]) and explained variance is defined as $100\times [1-\frac{RM{S}^{2}(CTW-SSLA)}{RM{S}^{2}(SSLA)}]$. (**b**) Grey shading (red contours) show the lag-correlation between subseasonal EKW_1_ averaged within [5°W–5°E; 1°S–1°N] and coastal SSLA (CTW_1_) as a function of the latitude and lag (day). (**c**,**d**) are similar to (**a**,**b**) for ROMS^EQ^. Note that to subtract the mesoscale variability, time-series are preliminarily smoothed using a 2°-width latitudinal running average filter. The figure has been realized using the Ferret program (http://ferret.pmel.noaa.gov/Ferret/).

The mean contribution of the 3 gravest CTW modes to the coastal SSLA variability is estimated for ROMS^CR^ over the 1993–2008 period (Fig. 2a). Results show that the second CTW mode (CTW_2_) dominates the coastal SSLA variability north of 12°S. Within [5°S–10°S], CTW_2_ Root Mean Square (RMS) is larger than 1 cm, explaining more than 55% of the coastal SSLA variability. South of 10°S, the amplitude of CTW_2_ drastically decreases. Further south, from ~13–15°S, the CTW_1_ becomes the most energetic regional-scale process and its contribution increases with latitude. Within [20°S–25°S] ([25°S–30°S]), CTW_1_ explains more than 75% (90%) of the coastal SSLA variability, with an RMS larger than 1.4 (1.6) cm. This alternation of the dominant CTW mode contributions confirms and extends the conclusions of *Illig et al*.^6^ over a longer period.

North of 15°S, where the alongshore subseasonal wind-stress variability remains weak (*cf*. Figure 10 in *Illig et al*.^6^), ROMS^EQ^ gravest CTW mode contributions to SSLA closely resemble the one of ROMS^CR^ (Fig. 2c). South of this latitude, and in particular south of 18°S, CTW_1_ subseasonal variability is notably weaker in ROMS^EQ^ compared to ROMS^CR^. Within [20°S–30°S], CTW_1_ subseasonal activity is lower than 0.8 cm RMS, i.e. almost 2 times less than in ROMS^CR^ (Fig. 2a). This is in agreement with the results of *Illig et al*.^6^ which showed that in the BUS, the alongshore wind-stress preferentially forces CTW_1_. However, even in the absence of subseasonal coastal wind forcing, CTW_1_ remains the dominant process behind the subseasonal regional coastal variability in the BUS (Fig. 2c), with an explained variance larger than 72% within the [20°S–30°S] coastal band.

Due to the design of ROMS^EQ^ experiment and recalling the weak impact of modal scattering for the gravest CTW mode in the BUS and equatorward (*cf*. Figure 7cd in *Illig et al*.^6^), the remote first baroclinic EKW is the dominant forcing mechanism of CTW_1_ propagations. Figure 2b,d illustrate the coherence between the EKW_1_ forcing averaged in the Eastern Equatorial Atlantic (EEA) and the coastal SSLA/CTW_1_ (shades of grey/red contours) along the southwestern African coast, for ROMS^CR^ and ROMS^EQ^ respectively. This diagnostic is based on a simple correlation analysis at each latitude, allowing a lag for the coastal propagations. Results show that ROMS^EQ^ coastal SSLA variability in the BUS ([18°S–32°S]) is unambiguously connected to the EKW_1_ activity, with statistically-significant maximum correlation larger than 0.45 when EKW_1_ leads the coastal SSLA by ~14–17 days (Fig. 2d). The sloping pattern is consistent with fast propagations with an estimated phase speed of ~4.5 m.s^−1^. As highlighted by the coinciding maximum lagged-correlation between EKW_1_ and CTW_1_, this coastal variability is predominantly explained by the propagation of the remotely-forced CTW_1_ (with theoretical phase speed of 5.5 m.s^−1^ ^5^). In ROMS^CR^ (Fig. 2b), when including the impact of the subseasonal coastal wind activity, correlations are weaker but the signature of the equatorially-forced CTW_1_ on the coastal SSLA variability on the BUS remains clear, with statistically-significant lag-correlations larger than 0.2 at lag +15–18 days. Our results imply that the oceanic equatorial connection, associated with the transmission of EKW_1_ into fast and weakly-dissipative CTW_1_ can impact the subseasonal variability down to the BUS, which challenges the findings of *Polo et al*.^1^, *Goubanova et al*.^15^ and *Illig et al*.^6^ (recalled in Fig. 1a).

Notably, north of 20°S, the pattern of lag-correlation between EKW_1_ and coastal SSLA differs from the straight path of CTW_1_ propagations. Within [10°S–20°S], it reveals a propagative pattern of statistically-significant correlation (>0.25) associated with larger lags in both simulations (Fig. 2b,d), with a weaker slope (1–1.5 m.s^−1^) than CTW_1_ propagations (~5 m.s^−1^). To determine the processes implicated and understand why previous studies have not depicted the signature of the equatorial connexion in the BUS, we now examine the characteristics of the remote equatorial forcing.

### Forcing and timing of the equatorial connection

**Figure 3:**
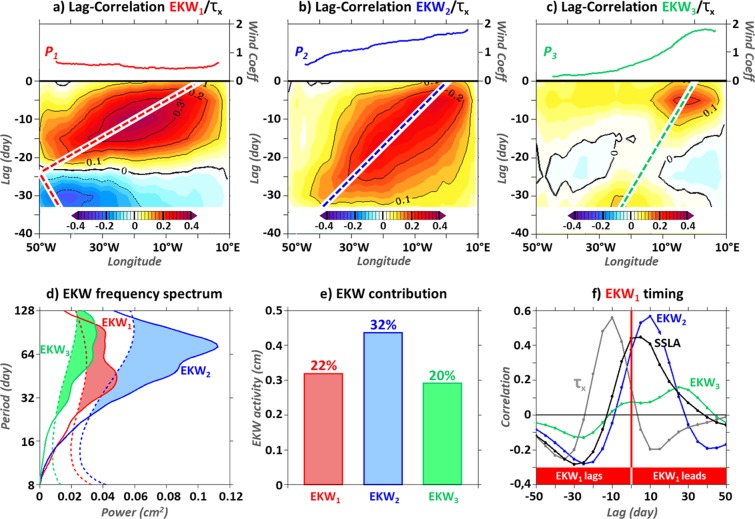
Mean (1993–2008) subseasonal EKW (averaged within [5°W–5°E; 1°S–1°N]) characteristics. (**a–c**) Lag-correlation (shading) between EKW and equatorial zonal wind-stress (averaged within [3°S–3°N], labelled τ_x_) as a function of longitudes and lags (day, left scale) for modes 1, 2, and 3 respectively. Negative lags indicate that wind-stress leads EKW. Dashed lines denote EKW propagation paths with phase-speeds^25^ of 2.5, 1.4, and 0.9 m.s^−1^. Wind projection coefficients are displayed above (right y-axis scale) as a function of longitudes. (**c**) Global Normalized Wavelet Power Spectrum^15^ of EKW modes 1, 2 and 3 (plain line, cm^2^) in red, blue, and green respectively. Dashed lines indicate the 95% confidence level and shadings underline significant values. (**e**) Gravest EKW RMS (cm), with explained variance relative to the SSLA (averaged within [5°W–5°E; 1°S–1°N]) specified above the bar-charts (%). (**f**) Lag-correlation between EKW_1_ and equatorial zonal wind-stress (τ_x_, averaged within [30°W–10°W; 3°S–3°N]; grey), EEA SSLA (black), EKW modes 2 and 3 (blue and green). Positive lags indicate that EKW_1_ leads. The figure has been realized using the Ferret program (http://ferret.pmel.noaa.gov/Ferret/).

Subseasonal EKW are triggered by subseasonal equatorial zonal wind-stress fluctuations, which are more energetic in the western basin (not shown). The magnitude of EKW results from the summation of the wind-forcing contributions accumulated retrospectively along the wave propagation/reflection path. In agreement with the EKW decay-scale and the magnitude of the wind-projection coefficient (*P*_*n*_) along the equator^25^ (Fig. 3a–c), each EKW mode is forced in different regions along the equatorial waveguide. In the EEA, EKW_1_ captures preferentially the equatorial zonal wind-stress fluctuations of the central basin [30°W–10°W] (Fig. 3a and grey line in Fig. 3f), and the higher the mode order, the more eastward EKW are forced (Fig. 3b,c). As a result, and in agreement with the solution of the OLM (not shown), EKW_1_ variability grasps the 1–2 month^−1^ equatorial zonal wind-stress fluctuations present in the western and center basin, while EKW modes 2 and 3 capture more the lower-frequency (2–4 month^−1^) zonal wind-stress variability of the Gulf of Guinea (Fig. 3d). Furthermore, the intensity of *P*_*n*_ concomitant with the region of high equatorial zonal wind-stress variability yields a dominant second EKW mode (EKW_2_) in the EEA, with substantially weaker contributions of EKW modes 1 and 3 (Fig. 3e), consistent with *Illig et al*.^6^. EKW_2_ explains 32% of the SSLA averaged within [5°W–5°E; 1°S–1°N], with a maximum correlation coefficient larger than 0.7 at lag 0. Individual contribution of EKW_1_ and EKW_3_ accounts for less than 22% of the EEA SSLA variability (Fig. 3e). Since EEA EKW_1_ is forced upstream EKW_2_ (Fig. 3a,b), EKW_1_ leads EKW_2_ by 10 days, with a correlation of 0.5 (blue line in Fig. 3f). Similarly, EKW_1_ leads EKW_3_ by 25 days (green line in Fig. 3f). Hence, due to the large spatial scales of the equatorial wind-stress forcing, the gravest EKW modal contributions are tangled and overshadowed by the variability of the dominant EKW_2_ (Fig. 3e).

As a consequence, north of 10°S, the dominant contribution of equatorially-forced CTW_2_ (Fig. 2a,c) is also correlated with the EKW_1_ contribution in the EEA: their correlation equals 0.38 when EKW_1_ (averaged within [5°W–5°E; 1°S–1°N]) precedes ROMS^CR^ CTW_2_ (averaged over [0°N–10°S; 0.5°-width coastal band]) by 25 days. This explains why the lag-correlation analysis between EKW_1_ and coastal SSLA variability (Fig. 2b,d) also captures, in addition to CTW_1_ propagations, the imprint of the slower-propagating higher-order CTW modes, with a velocity ranging between CTW_2_ and CTW_3_ phase speeds (i.e. 1.7 m.s^−1^ and 1.0 m.s^−1^, respectively; values averaged within [5°S–12°S] from Figure 4 in *Illig et al*.^5^). It corresponds to the secondary maximum of correlation that strays from the CTW_1_ path north of 15°S. Note that, the correlation analysis between EKW_2_ and CTW_1_ along the coast (not shown) reveals that the southward extension of positive correlations at lag ~ +40 days in the 15°S–30°S coastal band in Fig. 2b,d (not statistically-significant but consistent in space) corresponds to the scattering of CTW_2_ into CTW_1_.

**Figure 4:**
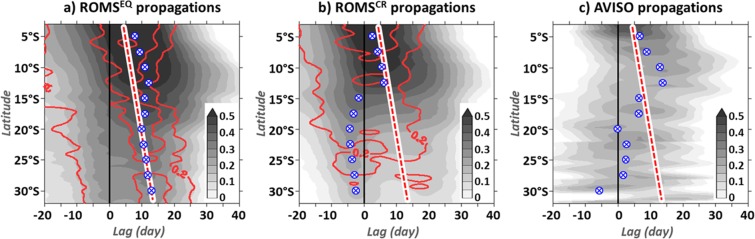
Same as Fig. 1b,d for lag-correlations between EEA SSLA and coastal SSLA/CTW_1_ (shadings/contours) using ROMS^EQ^ (**a**), ROMS^CR^ (**b**), and AVISO data^19^ (**c**). Blue dots indicate the lag of maximum correlation between EEA and coastal SSLA in function of the latitude (every 2.5°). Red dashed lines are the least-squares best-fit straight lines passing through the maximum correlation between ROMS^EQ^ EEA SSLA and CTW_1_ at each latitude. The altimeter product^19^ was produced by Ssalto/Duacs and distributed by Aviso+, with support from Cnes (https://www.aviso.altimetry.fr). The figure has been realized using the Ferret program (http://ferret.pmel.noaa.gov/Ferret/).

Due to the scarcity of the comprehensive sub-surface measurements, it is not possible to disentangled EKW modal contributions to the equatorial variability using observational data. The equatorial forcing is usually estimated using a proxy based on SLA averaged in the EEA^1,4,6,15^. OLM solutions (not shown) and SODA decomposition show that the EEA SSLA and its EKW_1_ contribution share some characteristics but are phase-shifted. Their lag-correlation is high (>0.4) when EKW_1_ contribution leads SSLA fluctuations by 0–10 days (black line in Fig. 3f). This is due to the fact that in the Gulf of Guinea, the modest EKW_1_ contribution (Fig. 3e) to SSLA is dwarfed by the dominant EKW_2_ variability that peaks 10 days after the passage of the fast EKW_1_ propagations (blue line in Fig. 3f). As a result, the lag-correlation between EEA SSLA and coastal SSLA in ROMS^EQ^ (Fig. 4a) shows a pattern resembling the one in Fig. 2d but shifted backward in time by ~5 days. The signature of the fast remotely-forced CTW_1_ down towards the BUS is clear in both CTW_1_ contribution (contours) and coastal SSLA fluctuations (shading), occurring only ~10 days after EEA SSLA are detected. North of 20°S, the broadening of the statistically-significant correlation pattern toward larger lags than the one associated with CTW_1_ denotes the signature of the remotely-forced slower higher-order CTW modes which dissipate north of 15°S^6^ (as illustrated on Fig. 1b). As a result, the lags associated with the maximum correlation between EEA and coastal SSLA (blue dots in Fig. 4a) show a transition at ~13°S, where they cease to increase monotonically with latitude. This feature is further amplified when the subseasonal local atmospheric forcing is at work. In ROMS^CR^ (Fig. 4b) and in agreement with altimetric observations (Fig. 4c), the equatorial connection seems to occur earlier in the BUS than north of the ABFZ and is almost in phase with the EEA SSLA variability. This may explain why the connection with the equatorial variability at subseasonal time-scales has not been documented in previous studies, possibly because the correct delay between the equatorial and the coastal SSLA variabilities was not identified.

In ROMS^CR^, the coastal wind activity dampens the connection between equatorial and coastal SSLA in the BUS (Fig. 4b), especially along the path of CTW_1_, in fair agreement with AVISO data (Fig. 4c). To further highlight the subseasonal equatorial connection in the BUS, in the next section we seek for periods of energetic EKW_1_-CTW_1_ propagations whose signature on the coastal SSLA variability can outweigh the imprint of the local forcing.

### Interannual modulation of the equatorial connection and coastal winds

**Figure 5:**
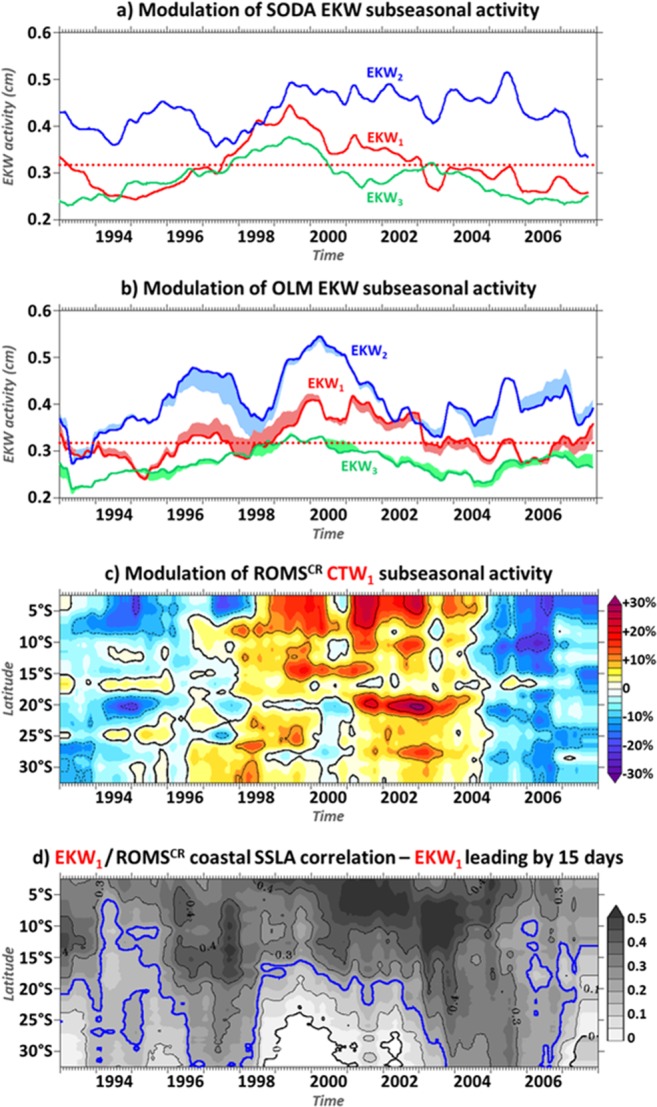
Interannual modulation of subseasonal EKW and CTW_1_. (**a**) 2-year running RMS of SODA EKW_1_, EKW_2_, and EKW_3_ (averaged within [5°W–5°E; 1°S–1°N], cm) in red, blue, and green respectively. Red dotted line indicates the mean (1993–2008) level of EKW_1_ activity. (**b**) Same as a) for OLM solution using mean (1993–2008) wave parameters (plain lines). Shadings highlight the rectifications associated with low-frequency time-varying OLM parameters. (**c**) Ratio (%) between the 2-year running RMS and the mean (1993–2008) RMS of ROMS^CR^ CTW_1_ (averaged within the 0.5°-width coastal band). (**d**) 2-year running correlation between EKW_1_ and ROMS^CR^ coastal SSLA, with EKW_1_ leading by 15 days. The figure has been realized using the Ferret program (http://ferret.pmel.noaa.gov/Ferret/).

We analyze the modulation of the equatorial forcing, focusing on EKW_1_, which is weakly dissipative when transmitted along the coast of southwestern Africa^6^ and propagate farther south than higher-order modes (Fig. 2). Figure 5a presents the interannual modulation of SODA gravest EKW modal contribution averaged in the EEA. Results show that the subseasonal EKW_1_ activity (2-year running RMS) is stronger during 1999–2002, with an RMS exceeding 0.36 cm. In 1999, the subseasonal EKW_1_ 2-year running RMS peaks to 0.45 cm, *i*.*e*. 40% more energetic than the mean (1993–2008) EKW_1_ activity (Fig. 3e). But EKW_1_ remains continuously overshadowed by the dominant EKW_2_ throughout the period of analysis (Fig. 5a) and explains less than 16% of the EEA SSLA variability over 1999–2002. The fair agreement with the simplified dynamics of the OLM forced using mean (1993–2008) wave parameters (*viz*.: phase-speed, dissipation, and *P*_*n*_; plain lines in Fig. 5b) indicates that the long EKW modulation is primarily driven by the modulation (in terms of magnitude and location) of the equatorial zonal wind-stress forcing. Note that the interannual modulation of the equatorial stratification, estimated through OLM solutions with low-frequency time-varying parameters^26^, accounts for 27% of the EKW_1_ interannual modulation (shadings in Fig. 5b) and agrees better with SODA EKW_1_ modulation (Fig. 5a).

In agreement with the interannual modulation of the remote equatorial forcing, CTW_1_ undergo a substantial modulation at interannual time-scales (Fig. 5c), with CTW_1_ noticeably more energetic when EKW_1_ activity is stronger in the EEA. As expected, this coherence is slightly weaker in ROMS^CR^ than in ROMS^EQ^ (not shown) in particular in the BUS, due to the locally-forced CTW_1_ contribution. Figure 5d further illustrates the interannual modulation of the coherence between the EEA EKW_1_ and coastal SSLA for ROMS^CR^ based on their 2-year running correlation. For this diagnostic, the equatorial forcing leads the coastal variability by 15 days, consistently with the timing of the CTW_1_ propagations in the BUS presented in Fig. 2b. Surprisingly, the period of strongest EKW_1_ activity (1999–2002) matches the period during which the coherence between the equatorial and the coastal subseasonal variabilities is the lowest in the BUS. During 1999–2002 and south of 20°S, the correlation remains lower than 0.2, *i.e*. below the level of statistical significance (blue line in Fig. 5d). Conversely, in 1997 and in 2004–2006 the correlation between EKW_1_ forcing and coastal SSLA is high and significant all along the southwest African coast, even though these periods do not correspond to particularly strong remote forcing episodes (Fig. 5a). Yet, the modulation of the connection between the equatorial and the coastal variability at interannual time-scales in ROMS^CR^ is not in agreement with ROMS^EQ^ solution (not shown). This calls for further investigation of the subseasonal local atmospheric forcing and its interannual modulation.

**Figure 6:**
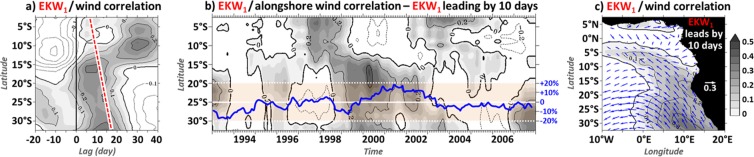
Interannual modulation of the subseasonal coastal alongshore surface winds. (**a**) 1999–2002 lag-correlation between EKW_1_ (averaged within [5°W–5°E; 1°S–1°N]) and alongshore wind (averaged within the 2°-width coastal band). The red dashed line is the least-squares best-fit passing through the maximum correlation between EKW_1_ and ROMS^CR^ CTW_1_ (Fig. 1b) at each latitude. (**b**) 2-year running correlation between EKW_1_ and coastal alongshore wind with EKW_1_ leading by 10 days. The encapsulated time-series shows the ratio (%) between the 2-year running RMS and the mean (1993–2008) RMS of the alongshore wind averaged within [20°S–30°S]. (**c**) Shading (arrow) shows the 1999–2002 correlation between EEA EKW_1_ and meridional (zonal and meridional) surface winds with EKW_1_ leading by 10 days. The figure has been realized using the Ferret program (http://ferret.pmel.noaa.gov/Ferret/).

Over 1999–2002, the lag-correlation between the subseasonal EKW_1_ in the EEA and the coastal alongshore surface wind (Fig. 6a) along the southwest African coast reveals a patch of statistically-significant covariability from 13°S to 34°S with EKW_1_ leading alongshore surface wind by 10–15 days. This coherence between downwelling EKW_1_ and upwelling-favorable winds is lined up with the path of CTW_1_ propagations (Fig. 2b) and reaches a maximum ~5 days before the peak in CTW_1_. Figure 6b further shows that, south of 15°S, the EKW_1_/alongshore wind connection (with EKW_1_ preceding alongshore wind by 10 days) is modulated at interannual time-scales. It is maximum (minimum) in 1999–2002 (1997 and 2004–2006). This is in fair agreement with the modulation of the subseasonal alongshore surface wind activity in the BUS (encapsulated time-series in Fig. 6b) and also consistent with the modulation of the EKW_1_ forcing (Fig. 5a) and its connection with the coastal SSLA variability (Fig. 5d). This suggests that there is a connection between the forcing of the EKW and the surface wind circulation along the southwest African coast at subseasonal time-scales that shapes the maximum latitude at which the equatorial dynamics imprints the coastal SSLA variability in the BUS (Fig. 5d). Figure 6c illustrates the positive correlation between the remote EKW_1_ and the meridional surface winds (10 days after) that breaks the link between the equatorial and the coastal SSLA variability along Angolan and Namibian coasts over 1999–2002. In the Gulf of Guinea, the wind pattern is favorable to the generation of downwelling EKW of second and higher-order modes (Fig. 3b,c) that dominates the EEA SSLA. As a result, the link between EEA SSLA and coastal SSLA is substantially weakened in the BUS 5 days before the passage of CTW_1_, splitting the bell-shaped correlation pattern (Fig. 4a) into a bi-modal profile (Fig. 4b). Finally, Figs 5d and 6 further suggest that the EEA zonal wind-stress that triggers the remote EKWs and the coastal wind along the coasts of Angola and Namibia are concomitantly modulated by the interannual variability of the basin-scale atmospheric circulation.

## Conclusions

**Figure 7:**
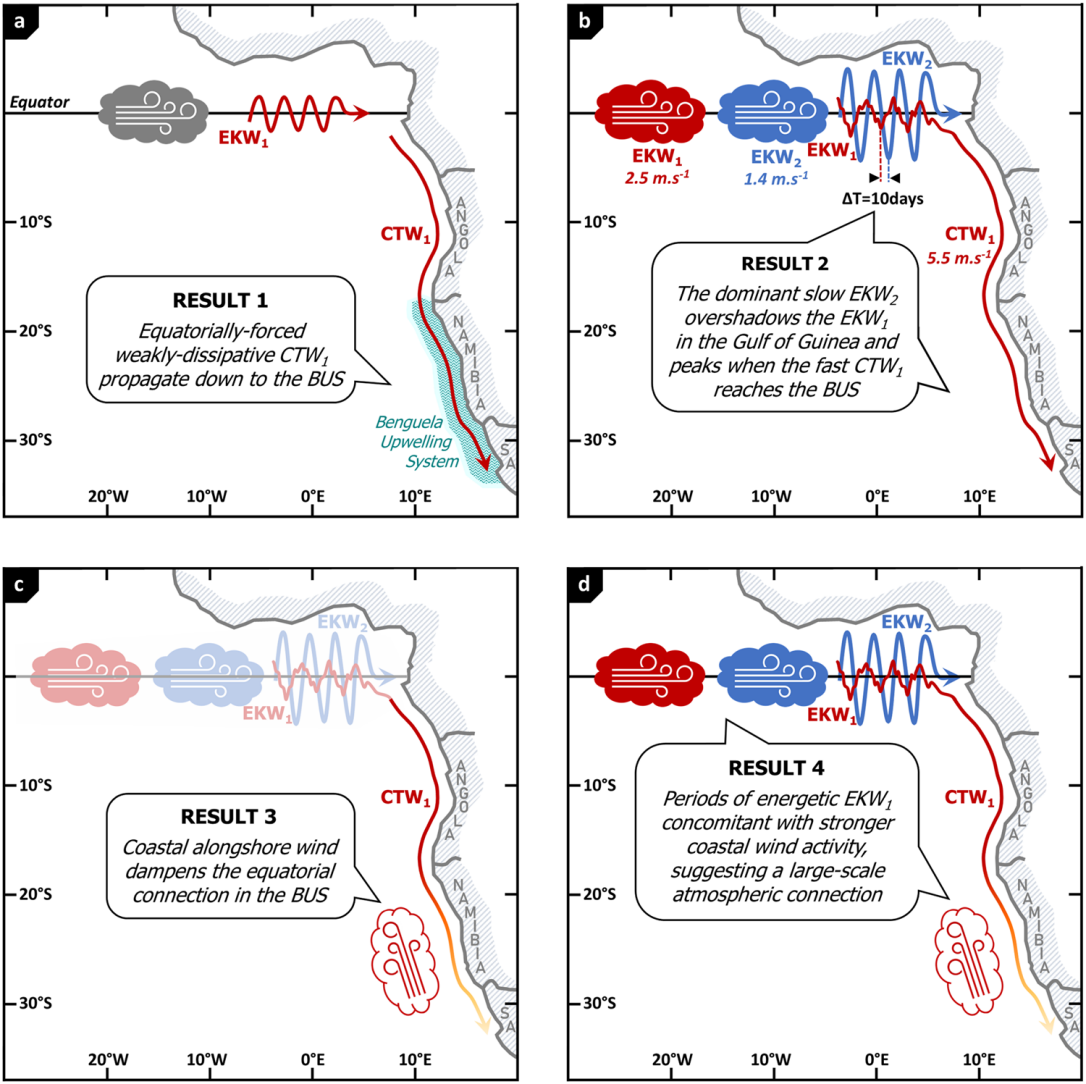
Set of schematics illustrating the main results. Panel (a) shows the equatorially-forced CTW_1_ propagating down to the BUS, as shown in section #3.1. Panel (b) emphasizes the characteristics of the equatorial forcing in the EEA obtained in section #3.2: the SSLA fluctuations are dominated by the contribution of EKW_2_, while the contribution of EKW_1_ that peaks 10 before EKW_2_ goes undetected. During this time frame, fast CTW_1_ reach the BUS, almost in phase with the SSLA fluctuations in the EEA. Panel (c) symbolizes the effects of the local atmospheric forcing in the BUS breaking the connection with the equatorial variability, as shown in sections #3.2 and #3.3. Panel (d) illustrates the results of section #3.3 and the possible large-scale atmospheric connection between the coastal wind activity and the EKW_1_ wind forcing. The figure has been realized using Microsoft PowerPoint (https://products.office.com/fr/powerpoint).

In this paper, we documented the connection between the equatorial variability and the coastal SSLA variability along the southwestern African coast at subseasonal timescales. Our main results are summarized in Fig. 7. We showed that the equatorially-forced weakly-dissipative CTW_1_ propagate down to the BUS (Fig. 7a), where they can impact the local marine ecosystem balance. Their forcing, the EEA EKW_1_ contribution, remains hidden by the dominant contribution of slower EKW_2_ that peaks 10 days after the passage of EKW_1_ (Fig. 7b). As a consequence, the remotely-forced CTW_1_ trigger coastal SSLA variations in the BUS almost in phase with the EKW_2_ and SSLA episodes in the Gulf of Guinea. For forecasting purposes, the EKW_1_ contribution unfortunately cannot be monitored from the actual observational network. However, the EKW and CTW decompositions of regional model outputs appear to be skilful tools to unravel modal contributions. These modal decomposition techniques should be applied to operational models in order to track the remotely-forced CTW propagations and anticipate their impact on the Benguela ecosystem resources and on the regional climate.

We then investigated the interannual modulation of the maximum latitude at which the equatorial dynamics imprints on the coastal variability. Results showed that it is primarily controlled by the modulation of the alongshore surface wind subseasonal activity that dampens the equatorial connection (Fig. 7c), more than by the change in the magnitude of the EKW_1_ activity. We disclosed the existence of a large-scale atmospheric connection between the forcing of downwelling EKW in the equatorial Atlantic and the upwelling-favorable alongshore wind in the BUS. Periods of energetic EKW_1_ are concomitant with stronger coastal wind activity that conceals the equatorial connection, as illustrated in Fig. 7d. This calls for further examination of the stressors controlling the low-frequency changes of the atmospheric surface circulation in the tropical Atlantic.

## Supplementary information

Model validation
