# Supplementary material for: Propagation of Subseasonal Equatorially-Forced Coastal Trapped Waves down to the Benguela Upwelling System
Source: Sci Rep. 2019 Mar 28;9:5306. doi: 10.1038/s41598-019-41847-1 (PMC6438976; doi:10.1038/s41598-019-41847-1)
Supplement: Supplementary file 1 — Model validation [file 41598_2019_41847_MOESM1_ESM.docx]

**Supporting Information for**

**“****Propagation of Subseasonal Equatorially-Forced Coastal Trapped Waves down to the Benguela Upwelling System “**

**Serena Illig**^1,2^ and **Marie-Lou Bachèlery**^2,3^

^1^ Laboratoire d’Etudes en Géophysique et Océanographie Spatiale (LEGOS),

CNRS/IRD/UPS/CNES, Toulouse, France

^2^ Department of Oceanography, MARE Institute, LMI ICEMASA,

University of Cape Town, Rondebosch, South Africa.

^3^ Nansen-Tutu Centre, Marine Research Institute, Department of Oceanography,

University of Rondebosch, South Africa.

**Contents**

1. Text S1 to S2
2. Figures S1 to S2

**Introduction**

This supporting information provides a validation exercise illustrating the realism of the models used to estimate the characteristics of the equatorial forcing and the Coastal Trapped Wave (CTW) contributions to the subseasonal variability in the southeastern Atlantic. Equatorial Kelvin Wave (EKW) and CTW contributions to Sea Level Anomalies (SLA) are inferred from the SODA reanalysis 2.1.6[^1^](#Carton_and_Giese_2008) and the ROMS Control Run experiment (ROMS^CR^), respectively. The use of global and regional models to derive EKW and CTW is motivated by their spatial and temporal coverage that allow to estimate their vertical structures and modal characteristics, which are not possible from the actual observation networks.

Note that SODA reanalysis has shown good skills for the study of the equatorial forcing at subseasonal and interannual time-scales in the tropical Atlantic[^2^](#Goubanova_et_al_2013)^-^[^7^](#Illig_et_al_2018b) and Pacific[^8^](#Dewitte_et_al_2008)^-^[^11^](#Vergara_et_al_2017)^,^ [^6^](#Illig_et_al_2018a)^,^ [^7^](#Illig_et_al_2018b) Oceans. Also, the ROMS southeastern Atlantic configuration used to derive the CTW characteristics closely resembles the one of *Bachelery et al*.[^4^](#Bachèlery_et_al_2016a)^,^ [^5^](#Bachèlery_et_al_2016b) and *Illig et al*.[^6^](#Illig_et_al_2018a)^,^ [^7^](#Illig_et_al_2018b), in which an extended evaluation of the southeastern Atlantic configuration performances is provided. The regional model reproduces realistically the mean state as well as the variability from subseasonal to interannual time-scales.

Then, in this document, we will only check the consistency between the model outputs and available observations in the southeastern Atlantic sector. We focus on the quantities that control the equatorial wave[^12^](#Illig_et_al_2004) and coastal wave[^6^](#Illig_et_al_2018a) characteristics, i.e., the mean structure along the equator, which controls the vertical mode structures, and the variability of surface zonal current and sea level, which also determine the EKW and CTW modal contributions.

The description of the associated figure follows below.

**S1: Realism of the SODA Reanalysis in the eastern equatorial Atlantic**

The realism of the SODA reanalysis is examined in the eastern equatorial Atlantic against the available *in-situ* PIRATA observations[^13^](#Bourles_et_al_2008), AVISO altimetric data[^14^](#Pujol_et_al_2016) and GlobCurrent[^15^](#Rio_et_al_2014) surface zonal current estimations (**Fig.S1ab**). **Figure S1a** shows the vertical profile of the temperature at [0°E-0°N] averaged over the 1998-2008 period for SODA (red line) and PIRATA subsurface data (green line) in the first 250 meters. Note that gaps occur at various times in 5-day PIRATA records due to vandalism or data failure. For consistency, these gaps have been replicated in the models timeseries. Despite a typical deeper (SODA 20°C isotherm (D20) is ~40 meters deeper than PIRATA) and too diffuse (the layer comprised between the 16°C and 24°C isotherms is thicker by 25%) thermocline, results indicate a fair representation of the mean vertical temperature structure in the Gulf of Guinea. In addition, **Figure S1b** provides a Taylor diagram[^16^](#Taylor_2001) that includes an evaluation of the fluctuations of the depth of the D20 (used as a proxy for the position of the equatorial thermocline) and the dynamic height anomalies against PIRATA data at [10°W-0°N] and [0°E-0°N]. Note that due to the gaps in PIRATA records, it was not possible to estimate subseasonal anomalies in the observations. We thus quantified the variability relative to the mean state. Beside the fact that SODA D20 fluctuations are substantially more energetic than in the observations, model and data agree well with Taylor scores (Eq 4 of *Taylor*[^16^](#Taylor_2001)) larger than 0.7. **Fig.S1b** further indicates the good statistical agreement between SODA variability and observations at subseasonal and interannual time-scales, when compared to remotely-sensed observations of SLA and Zonal Current Anomalies (ZCA) over 1993-2008. In the Gulf of Guinea and at both time-scales, SODA and observations share the same level of energy and are significantly and strongly correlated (r > ~0.7).

The good agreement between SODA and observations for these various parameters gives confidence in SODA reanalysis to capture the salient features of the propagating characteristics of EKW contributions in the eastern equatorial Atlantic.

**S2: Realism of ROMS^CR^ simulation in the southeastern Atlantic**

ROMS^CR^ skills in reproducing the mean vertical stratification in the eastern equatorial Atlantic and the subseasonal SLA variabilities along the path of the EKW and CTW propagations are evaluated. In **Figure S1a,** the comparison of the mean (1998-2008) temperature vertical profile in the model (blue line) with PIRATA observations (green line) highlights the realistic vertical structure of ROMS^CR^ along the equatorial waveguide. There is no significant bias in the mean sea surface temperature and the thermocline is less than 5 (10) meter deeper (too diffuse) in the regional model. **Figure S1c** further presents the model/observations comparison for subseasonal SLA time series at each longitude along the eastern equatorial sector (squares) over the 1993-2008 period using a Taylor diagram. Results show a very good consistency between model and altimetric data with an accurate estimation of the magnitude of the subseasonal activity and a correlation largely above the 99% confident level threshold[^17^](#Sciremammano_1979). Along the coast of southwestern Africa, subseasonal SLA are also in very good agreement with the observations with Taylor scores above 0.7. The marginally lower agreement in the Benguela upwelling system is most likely due to the strong local mesoscale activity whose features, in the absence of data assimilation, cannot agree in space and time with AVISO estimations.

In conclusion, the overall good agreement between ROMS^CR^ and observations implies that the linear equatorial dynamics is adequately constrained by SODA boundary forcing in the regional model and it is successfully transmitted along the southwestern coast of Africa at subseasonal time-scales.


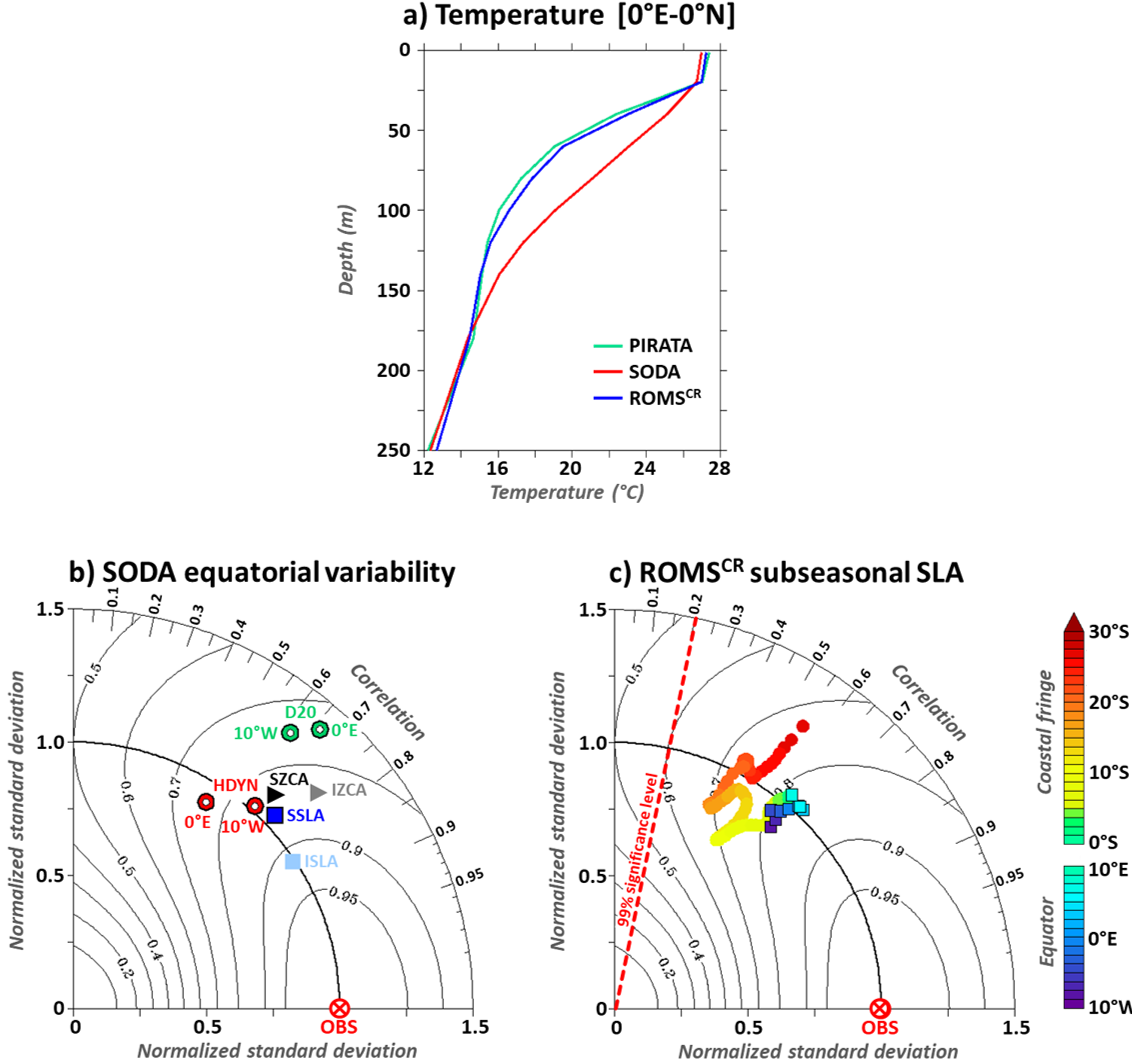


**Figure S1**: SODA and ROMS^CR^ performances. a) mean (1998-2008) vertical temperature profile (°C) at [0°E;0°N] for PIRATA data, SODA, and ROMS^CR^ outputs, in green, red, and blue lines respectively. b) Taylor diagram[^16^](#Taylor_2001) summarizing SODA skills compared to various observations datasets: 20°C isotherm (green circles, D20) and dynamic height (red circles, HDYN) at [0°N; 10°W and 0°E] from PIRATA data over the 1998-2008 period; SLA from AVISO (blue squares) and Zonal Current Anomalies (ZCA) of GlobCurrent (grey triangles) averaged within [3°N-3°S;5°W-5°E] over the 1993-2008 period for Subseasonal (S; dark shade) and Interannual (I; light shade) time-scales. c) Taylor diagram illustrating ROMS^CR^ skills in representing the altimetric subseasonal SLA along the equator (averaged within [1°S-1°N], squares) and along the southwestern coast of Africa (averaged within the 50-km coastal fringe, circles) over 1993-2008. Colors indicate the longitude along the equator and the latitude along the coast. Isolines provide a measure of the skill as defined by equation (4) from *Taylor*[^16^](#Taylor_2001)). Dashed radius line denotes the limit of the 99% significance level for the correlation[^17^](#Sciremammano_1979).

**References:**

**1.** Carton, J. A. & Giese, B. S. A reanalysis of ocean climate using simple ocean data assimilation (SODA), *Mon. Weather Rev*. **136**, 2999–3017 (2008).

**2.** Goubanova K., Illig, S., Machu, E., Garçon, V. & Dewitte, B. SST subseasonal variability in the Benguela upwelling system as inferred from satellite observations (2000-2008). *J. Geophys. Res.* **118**, 4092–4110 (2013).

**3.** Bachèlery, M.-L. Variabilité côtière physique et biogéochimique en Atlantique Sud-Est: rôle du forçage atmosphérique local versure téléconnexion océanique. *Ph.D. thesis*, 215 pp., Laboratoire d’Etude en Geophysique et Océanographie Spatiale (LEGOS), Univ. of Paul Sabatier, Toulouse, France (2016).

**4.** Bachèlery, M.-L., Illig, S. & Dadou, I. Interannual variability in the South-East Atlantic Ocean, focusing on the Benguela Upwelling System: Remote versus local forcing. *J. Geophys. Res*. **120**, 284-310 (2016).

**5.** Bachèlery, M.-L., Illig, S. & Dadou, I. Forcings of Nutrient, Oxygen and Primary Production interannual variability in the South-East Atlantic Ocean. *Geophys. Res. Lett*. **43**, 8617–8625 (2016).

**6.** Illig, S., Cadier, E., Bachèlery, M.-L. & Kersalé, M. Subseasonal coastal-trapped wave propagations in the southeastern Pacific and Atlantic Oceans: 1. A new approach to estimate wave amplitude. *J. Geophys. Res.* **123**, 3915-394 (2018).

**7.** Illig, S., Bachèlery, M.-L. & Cadier, E. Subseasonal coastal-trapped wave propagations in the southeastern Pacific and Atlantic Oceans: 2. Wave characteristics and connection with the equatorial variability. *J. Geophys. Res.* **123**, 3942–3961 (2018).

**8.** Dewitte B., Purca, S., Illig, S., Renault, L. & Giese, B. Low frequency modulation of the intra-seasonal equatorial Kelvin wave activity in the Pacific Ocean from SODA: 1958-2001. *J. Climate* **21**, 6060-6069 (2008).

**9.** Dewitte B., J. Vazquez-Cuervo, K. Goubanova, S. Illig, K. Takahashi, G. Cambon, S. Purca, D. Correa, D. Gutierrez, A. Sifeddine & L. Ortlieb (2012), Change in El Niño flavours over 1958-2008: Implications for the long-term trend of the upwelling off Peru. Deep Sea Research II, doi:10.1016/j.dsr2.2012.04.011.

**10.** Illig, S., Dewitte, B., Goubanova, K., Cambon, G., Boucharel, J., Monetti, F., Romero, C., Purca, S. & Flores R. Forcing mechanisms of intraseasonal SST variability off central Peru in 2000–2008. *J. Geophys. Res.* **119**, 3548–3573 (2014).

**11.** Vergara, O., Dewitte, B., Ramos, M. & Pizarro, O. Vertical energy flux at ENSO time scales in the subthermocline of the Southeastern Pacific. *J. Geophys. Res*. **122**, 6011–6038 (2017).

**12.** Illig, S., Dewitte, B., Ayoub, N., du Penhoat, Y., Reverdin, G., De Mey, P., Bonjean, F. & Lagerloef, G. S. E. Interannual long equatorial waves in the tropical Atlantic from a high-resolution ocean general circulation model experiment in 1981-2000. *J. Geophys. Res*. **109**, C02022 (2004).

**13.** Bourlès, B. et al., The PIRATA program. *Bull. Am. Meteorol. Soc*. **89**, 1111-1125 (2008).

**14.** Pujol, M.-I., Faugère, Y., Taburet, G., Dupuy, S., Pelloquin, C., Ablain, M. & Picot, N. DUACS DT2014: the new multi-mission altimeter data set reprocessed over 20 years. *Ocean Sci*. **12**, 1067-1090 (2016).

**15.** Rio, M.-H., Mulet, S. & Picot, N. Beyond GOCE for the ocean circulation estimate: Synergetic use of altimetry, gravimetry, and in situ data provides new insight into geostrophic and Ekman currents. *Geophys. Res. Lett*. **41**, 8918–8925 (2014).

**16.** Taylor, K. E. Summarizing multiple aspects of model performance in a single diagram. *J. Geophys. Res.* **106,** 7183–7192 (2001).

**17.** Sciremammano, F. A Suggestion for the Presentation of Correlations and Their Significance Levels. *J. Phys. Oceanogr*. **9**, 1273-1276 (1979).
